# MiRNAs in Extracellular Vesicles as Biomarkers in Plasma of Papillary Thyroid Cancer Patients: A Proof-of-Concept Study

**DOI:** 10.3390/biology13090743

**Published:** 2024-09-22

**Authors:** Giuseppa D’Amico, Radha Santonocito, Godfrey Grech, Giuseppa Graceffa, Calogero Cipolla, Federica Scalia, Samuele Raccosta, Mauro Manno, Everly Conway de Macario, Alberto J. L. Macario, Francesco Cappello, Francesca Rappa, Celeste Caruso Bavisotto, Claudia Campanella

**Affiliations:** 1 Section of Human Anatomy, Department of Biomedicine, Neuroscience and Advanced Diagnostics (BIND), University of Palermo, 90127 Palermo, Italy; giuseppa.damico01@unipa.it (G.D.); radha.santonocito@unipa.it (R.S.); federica.scalia02@unipa.it (F.S.); francesco.cappello@unipa.it (F.C.); francesca.rappa@unipa.it (F.R.); claudia.campanella@unipa.it (C.C.); 2 Department of Pathology, Faculty of Medicine and Surgery, University of Malta, MSD 2080 Msida, Malta; godfrey.grech@um.edu.mt; 3 Department of Precision Medicine in the Medical, Surgical and Critical Area, University of Palermo, 90127 Palermo, Italy; giuseppa.graceffa@unipa.it (G.G.); calogero.cipolla@unipa.it (C.C.); 4 Cell-Tech Hub, Institute of Biophysics, National Research Council of Italy, 90146 Palermo, Italy; samuele.raccosta@ibf.cnr.it (S.R.); mauro.manno@cnr.it (M.M.); 5 Department of Microbiology and Immunology, School of Medicine, University of Maryland at Baltimore-Institute of Marine and Environmental Technology (IMET), Baltimore, MD 21202, USA; econwaydemacario@som.umaryland.edu (E.C.d.M.); ajlmacario@som.umaryland.edu (A.J.L.M.); 6 Euro-Mediterranean Institute of Science and Technology (IEMEST), 90139 Palermo, Italy; 7 The Institute of Translational Pharmacology, National Research Council of Italy (CNR), 90146 Palermo, Italy

**Keywords:** thyroid cancer, extracellular vesicles, microRNAs, cancer biomarkers, chaperone system, Hsp60, CCT, liquid biopsy

## Abstract

**Simple Summary:**

Cancer diagnoses, including papillary thyroid carcinoma (PTC), are increasing, and early detection is critical for successful treatment. To help address this, our study focuses on identifying reliable biomarkers—specific molecules indicating the presence of disease—that can be detected through non-invasive methods like liquid biopsies. We examined small RNA molecules, known as microRNAs (miRNAs), found in microparticles called extracellular vesicles (EVs) in blood samples from PTC patients, which are linked to the development of PTC. Our results showed these miRNAs were present at higher levels in PTC patients and returned to normal after surgery, suggesting their potential as reliable indicators of PTC. The use of these biomarkers in EVs could allow easier, less invasive, and more frequent testing compared to current methods, improving early detection and monitoring of thyroid cancer, thus benefiting patients and healthcare systems.

**Abstract:**

Background: The incidence of various types of cancer, for example, papillary thyroid carcinoma (PTC), is on the rise. Since therapeutic success depends greatly on early diagnosis, reliable diagnostic biomarkers must be identified, and easy-to-apply tools for detecting them must urgently be standardized. Here, we contribute to solving this medical challenge by assessing miRNAs suspected of promoting carcinogenesis in extracellular vesicles (EVs) that can be routinely obtained via liquid biopsy. We profit from current progress in cancerology that provides innovations in liquid biopsy and EVs analysis, along with the identification of miRNAs and chaperone system (CS) components implicated in carcinogenesis. Methods: We measured in EVs obtained from circulating blood plasma from PTC patients the levels of three miRNAs implicated in thyroid cancer, hsa-miR-1-3p, hsa-miR-206, and hsa-miR-221-3p, and most likely involved in the regulation of two members of the CS, Hsp60 and CCT. EVs were isolated from the plasma of patients with PTC and controls with benign goiter (BG) and from the culture medium of a PTC cell line (MDAT32) and were appropriately characterized. Results: The levels of miRNAs determined by RT-qPCR were consistently higher in PTC patients and decreased down to control levels after thyroidectomy. Bioinformatics showed that the miRNAs target genes are associated with the molecular pathogenesis of PTC. Conclusions: Our exploratory study reaffirms the potential in clinics of the selected miRNAs in EVs as useful biomarkers of PTC easily accessible via liquid biopsy, which is minimally invasive and amenable to periodic repetition, an improvement compared to the established fine-needle aspirate biopsy.

## 1. Introduction

Thyroid cancer (TC) is a malignancy that has significantly increased in frequency worldwide in recent years, especially in the most developed countries [1]. Among the various types of thyroid cancer, papillary thyroid carcinoma (PTC) is the most common, predominantly affecting women compared to men with a 3:1 ratio [1]. The typical procedure for the initial evaluation of nodular thyroid disease involves cytopathological analysis by percutaneous fine needle aspiration biopsy. This procedure has limitations; it is invasive, time-consuming, and often inaccurate, particularly because it may yield false-negative results for large thyroid nodules and is not suitable for repeating applications for a tumor follow-up [2]. Thus, to minimize patient harm and enhance diagnostic precision and specificity, it is crucial to develop a non-invasive, well-tolerated method for identifying PTC. Liquid biopsy is a minimally invasive detection method with potential application to several diseases, including PTC. However, there are currently no specific biomarkers available for PTC. Liquid biopsy can provide extracellular vesicles (EVs) and represents a simpler, minimally invasive alternative to needle biopsy. EVs are small, membrane-bound nanoparticles secreted by almost all cell types in various fluids, including blood, urine, and saliva [3]. Their significant role in cancer biology is documented, with implications for tumor growth, metastasization, and the development of drug resistance and immune evasion [4,5,6]. Because of these findings, EVs have garnered considerable interest as potential cancer biomarkers. They possess various characteristics that make them promising candidates, for example: (i) EVs are shed from all cell types in the organism; (ii) molecules contained in EVs, including proteins, lipids, DNA, mRNAs, and microRNAs (miRNAs), are components of the cells from which the EVs originate and reflect their physiological or pathophysiological status; (iii) the molecular cargo of EVs can be influenced by their microenvironment [6,7,8]. Moreover, EVs in liquid biopsy are informative pathological specimens more easily accessible than tissue samples obtained by traditional methods such as needle aspiration or surgical biopsies.

Recent advances in cancer research have been achieved on various fronts, and one of these is the study of the Chaperone System (CS) [9,10]. The chief components of the CS are the molecular chaperones, some of which are called heat shock protein (Hsp). These are involved in many physiological mechanisms in normal cells and are present inside EVs secreted by cancer cells. Hsps released by cancer-derived EVs play a role in human cancer development and immune system stimulation, and their levels in the cancer cells may be modulated by specific miRNAs [11,12,13,14]. Based on these premises, chaperones associated with miRNAs in circulating EVs can be considered promising biomarkers for cancer diagnosis and patient monitoring.

In the past decade, there has been growing interest in the study of noncoding RNAs (ncRNAs), a class of RNA molecules that perform several regulatory functions within cells and include miRNAs, which are involved in the post-transcriptional regulation of gene expression. miRNAs are dysregulated in different types of human tumors, suggesting a role as tumor promoters or tumor suppressors. Because of this, the levels of miRNAs have been determined in various cancer types [7,13,14,15,16]. For example, the levels of circulating miRNAs in patients with thyroid cancer have been measured in a search for diagnostic biomarkers [7,17,18].

The study reported here focused on the levels of miRNAs contained within EVs, taking advantage of the fact that the EVs’ phospholipid bilayer protects RNA from circulating RNAses. We determined the levels of hsa-miR-1-3p, hsa-miR-206, and hsa-miR-221-3p (hereinafter miR-1-3p, miR-206 and miR-221-3p) in patients with papillary thyroid cancer (PTC) and compared them with the levels in a control group constituted of patients with benign goiter (BG). We focused on miR-1-3p and miR-206 because of their known involvement in the regulation of expression of the gene encoding the molecular chaperone Hsp60 [19], a protein implicated in the tumorigenesis of PTC [20,21,22], and we also focused on miR-221-3p because it is usually augmented in PTC [23,24,25,26]. The levels of these three miRNAs were measured in EVs extracted from the plasma of patients with PTC and BG both before and after surgical thyroidectomy. The data were also compared with those obtained from EVs isolated from the culture medium of a PTC cell line (MDA-T32).

Once the experimental data were obtained, a bioinformatics analysis was carried out to predict the target genes of the miRNAs examined to better understand the possible dysregulated pathways that lead to the development of thyroid neoplasia and tumor progression via metastatic dissemination.

The data obtained from plasma, culture medium, and bioinformatics analysis reinforced the idea that miR-1-3p and miR-206 are promising candidates as thyroid cancer-specific biomarkers, particularly considering their association with Hsp60 expression. In addition, analysis of miR-221-3p within EVs confirmed data in the literature, indicating that the levels of miRNAs in circulation are elevated in PTC patients.

## 2. Materials and Methods

### 2.1. Patients

The patients recruited for this study underwent surgery at the Department of Oncological Surgery of the University Hospital of Palermo Paolo Giaccone between 2020 and 2021. The Ethics Committee of University Hospital AUOP Paolo Giaccone of Palermo approved the study (Approval Number: N. 05/2017 of 05/10/2017) and was conducted in accordance with the Declaration of Helsinki. All patients gave their written informed consent. Twelve patients were studied, seven with papillary thyroid carcinoma (PTC) and five with non-toxic benign goiter (BG), the latter used as controls. Two blood samples were taken from each patient, one on the day of surgery before the operation (BS, before surgery) and a second (after surgery, AS) on the day of release from the hospital (1 week later).

### 2.2. Cell Culture

Human papillary thyroid carcinoma cells (MDA-T32 CRL-3351, ATCC) were grown in RPMI (Roswell Park Memorial Institute) 1640 medium (Gibco-Thermo Fisher Scientific, Waltham, MA, USA), supplemented with 10% heat-inactivated Fetal Bovine Serum (FBS), 100 U/mL penicillin, 100 g/mL streptomycin, 1× MEM Non-Essential Amino Acids solution (Gibco-Thermo Fisher Scientific, Waltham, MA, USA) and 2 mM L-glutamine, in a humidified 37 °C atmosphere containing 5% CO_2_.

### 2.3. EVs Isolation from Plasma

Four mL of whole blood was collected in EDTA tubes from each subject, and plasma was separated by centrifugation at 2000× *g* for 20 min to remove blood cells. Plasma samples were stored at −80 °C and processed within 3 months of storage. Subsequently, plasma was centrifuged at 1800× *g* for 30 min at 4 °C to remove cell debris and large particles. Afterward, it was diluted 1:1 with cold PBS (Phosphate-buffered saline) to reduce its viscosity and filtered with 0.22 μm pore filters. Next, the samples were ultracentrifuged at 110,000× *g* for 2 h at 4 °C. The supernatant was removed, and the pellet was resuspended in cold PBS and ultracentrifuged at 110,000× *g* for 2 h at 4 °C. Finally, the supernatant was discarded, and the pellet resuspended in two aliquots: (a) in 100 μL of radioimmunoprecipitation assay (RIPA) buffer for Western Blot analysis (the entire procedure was carried out on ice to prevent protein degradation; and (b) in 100 μL of PBS for miRNA extractions or biophysical characterization of EVs [27].

### 2.4. EVs Isolation from Cell Culture Medium

When MDA-T32 cells in culture reached 80% confluence, they were starved by growing them in a medium without FBS for 24 h. Afterward, a cell culture medium was collected for EVs isolation. Fifteen ml of cell culture medium were first centrifuged at 800× *g*, for 10 min at 4 °C, to remove dead cells; then, the supernatant was recovered and further centrifuged at 13,000× *g* for 20 min at 4 °C to remove cell debris and mitochondrial contaminants. At the end of this step, the supernatant was collected and filtered through a 0.22 μm pore filter. The filtered supernatant was ultracentrifuged for 2 h, at 110,000× *g* at 4 °C. After ultracentrifugation, the supernatant was discarded, while the EV-pellet was collected and resuspended in 100 μL of RIPA buffer for Western blot analysis, or in 100 μL of PBS for miRNA extractions or biophysical characterization of EVs. Experiments were performed in triplicate.

### 2.5. EVs Characterization

#### 2.5.1. NanoSight™ Particle Tracking Analysis

Nanoparticle size distribution and concentration were measured using a NanoSight NS300 instrument (Malvern Panalytical Ltd, Cambridge, UK). The instrument was equipped with a 488 nm laser, a high-sensitivity sCMOS camera, and a syringe pump. EV samples were resuspended in PBS and then diluted in particle-free PBS (filtered through 20 nm filters) to generate a dilution in which 20–120 particles per frame were tracked to obtain a concentration within the recommended measurement range (1–10 × 10^8^ particles/mL). Five experiment videos of 60-s duration were analyzed using NTA 3.4 Build 3.4.003 (camera level 15–16) (Malvern Panalytical Ltd, Cambridge, UK).

#### 2.5.2. Size Distribution Determined by Dynamic Light Scattering (DLS)

EV samples were pipetted after thawing and centrifuged at 1000× *g* for 10 min at 4 °C to remove eventual aggregates or dust. The supernatant was maintained at 20 °C in a temperature-controlled cell compartment of a Brookhaven Instruments BI200-SM goniometer equipped with a He-Ne laser (JDS Uniphase 1136P) (Brookhaven Instruments, Nashua, NH, USA) tuned at 633 nm and a Hamamatsu single-pixel photon counting module (mod. C11202-050) (Brookhaven Instruments, Nashua, NH, USA). Dynamic Light Scattering (DLS) measurements were carried out as previously described [28]. The size distribution of vesicles is calculated by assuming that the diffusion coefficient distribution is shaped as a Schultz distribution, which is a two-parameter asymmetric distribution, determined by the average diffusion coefficient $\bar{D}$ (σ_z_ is the diameter corresponding to $\bar{D}$) and the polydispersity index PDI [28,29,30]. The analysis was performed using two species: the former to consider the contribution from small particles around 20 nm or less (proteins or small molecules), the latter for the contribution assigned to vesicles.

#### 2.5.3. Scanning Transmission Electron Microscopy (STEM)

The purified EVs were resuspended in PBS. Then, the EV preparations were mounted on a formvar nickel grid (200 mesh squares grid) by layering the grids over 10 μL drops of EV preparations to allow adherence of particles to the grids, and they were incubated overnight at 24 °C. Grid-mounted preparations were prepared for contrast staining by treating them with 1% uranyl acetate (*w*/*v*) for 5 min. After washing with methanol twice for 5 min, a filter paper was used to blot away the excess liquid from the grids, and then the grids were treated with Reynold’s solution for 3 min [27]. Finally, the grids were rinsed in distilled water twice for 5 min. After this procedure, the grids were ready to be observed and photographed under a scanning electron microscope (SEM) FEI-Thermo Fisher Versa 3D equipped with a retractable STEM detector (Therno Fisher, Waltham, MA, USA).

### 2.6. Protein Extraction, Quantification, and Western Blot Analysis

Western blot was performed to detect the presence of typical EV-associated proteins to confirm the presence of EVs in the biological sample. EVs obtained by ultracentrifugation were lysed using radioimmunoprecipitation assay (RIPA) buffer containing 50 mM Tris/HCl, 150 mM NaCl, 1% NP-40, 1 mM EGTA and supplemented with Protease Inhibitor Cocktail (Sigma-Aldrich, St. Louis, MO, USA) (1 mM AEBSF, 1 μM Aprotinin, 50 μM Bestatin, 15 μM E-64, 20 μM Leupeptin, 10 μM Pepstatin A). The procedure was carried out on ice to prevent protein degradation. EV lysates were then homogenized by pipetting up and down several times on ice for 1 h. To obtain only the whole protein suspension, lysates were centrifuged at 16,000× *g* for 20 min. The supernatant was collected and stored in new tubes at –20 °C. The total protein concentration present on EVs was determined by using the Bradford assay, and a standard Western blot procedure was performed using the following antibodies: anti-CD81 (Santa Cruz Biotechnology, Dallas, TX, USA, sc-70803); anti-Alix (Santa Cruz Biotechnology, sc-53538); anti-Calnexin (Santa Cruz Biotechnology, sc-46669). Each experiment was performed at least three times.

### 2.7. miRNA Extraction, Retro Transcription, and Real-Time PCR (qPCR)

miRNAs from EVs isolated from the cell culture medium and plasma samples of patients were obtained using the miRNeasy Mini Kit (QIAGEN, Hilden, Germany), according to the manufacturer’s instructions. The concentration of the miRNA-enriched fractions was determined using a Thermo Scientific NanoDrop 2000 1-position Spectrophotometer (Thermo Scientific, Waltham, MA, USA). cDNA was synthesized using miRCURY LNA RT Kit (QIAGEN, Hilden, Germany) and the Applied BiosystemsTM VeritiTM 96-Well Thermal Cycler (Fisher Scientific, MA, USA). Afterward, Real-Time PCR was performed by using the miRCURY LNA SYBR^®^Green PCR Kit (QIAGEN, Hilden, Germany). Reactions were carried out by adding primers specific for human miR-1-3p, miR-206, miR-221-3p, and miR-16-1-3p, the latter was used as a housekeeping internal control (Table 1).

Real-time PCR assays were performed on the real-time PCR cycler Rotor-Gene Q (QIAGEN). The miRNA levels were normalized to the levels obtained for miR-16-1-3p, used as housekeeping control. Changes in the transcript level were calculated using the 2^-ΔΔCt^ method. Experiments were performed in triplicate.

### 2.8. Bioinformatic Analysis

To validate the data obtained experimentally, a bioinformatics analysis was performed to better understand the potential significance of our findings. The analysis, performed on TargetscanHuman 8.0 (https://www.targetscan.org/vert_80/; accessed on 10 June 2024) and then supported by the data in the literature on Pubmed, predicts the targets of miRNAs that were found to be upregulated in thyroid cancer and specifically in PTC.

### 2.9. Statistical Analysis

Statistical analyses of miRNA expression levels were performed using the GraphPad Prism 8.8 package (GraphPad Inc., San Diego, CA, USA). Comparisons were performed using one-way ANOVA with Tukey’s multiple comparison test for plasma data. On the other hand, for data from the cell line, statistical analysis was performed using the unpaired two-tailed Student’s *t*-test. All data are presented as mean ± SD, and the level of statistical significance was set at *p* ≤ 0.05.

## 3. Results

### 3.1. Characterization of Isolated EVs

**Figure 1:**
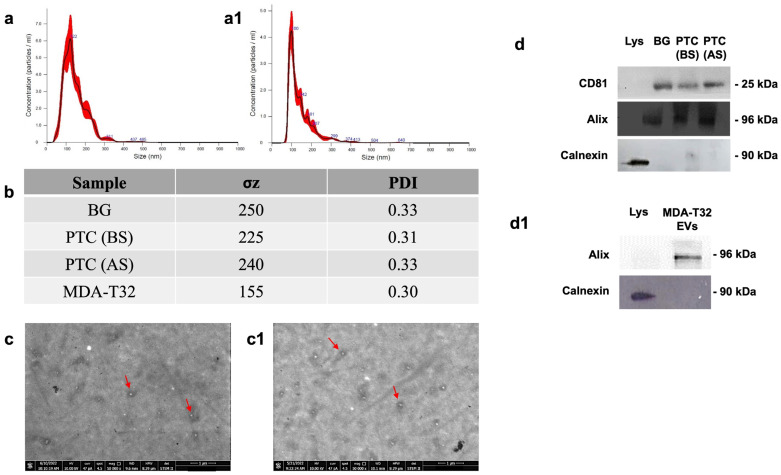
EVs characterization. (**a**) NTA measurement of EVs isolated from plasma samples of patients with PTC. EVs samples were resuspended and diluted in particle-free PBS (filtered by 20 nm filters). NTA profile of EVs shows the mean and mode size of EVs. The mode particle size of ~124 nm confirmed the presence of EVs. (**a1**) NTA measurement of EVs isolated from cell culture medium of MDA-T32 cell line. EVs samples were resuspended and diluted in particle-free PBS (filtered by 20 nm filters). NTA profile of EVs shows the mean and mode size of EVs. The mode particle size of ~100 nm confirmed the presence of EVs. (**b**) Dynamic Light Scattering (DLS) results for the size distribution of EVs obtained from plasma samples of patients with BG, patients with PTC before (BS) and after surgery (AS), and obtained from MDA-T2 cell culture medium. Parameters of biophysical characterization of vesicles by DLS (σ_z_: size distribution; PDI: polydispersity index). The size distribution shows that there are EVs in the sample. (**c**,**c1**) Representative images of EVs isolated from plasma samples of patients with papillary thyroid carcinoma (**c**) and from cell culture medium of MDA-T32 cell line (**c1**) obtained with a scanning electron microscope (SEM) FEI-Thermo Fisher Versa 3D. Scale bar 1 µm. 50,000× magnification. (**d**) Representative immunoblotting detection of CD81, Alix, and Calnexin in internal control cell lysate (Lys) and EVs isolated from plasma samples before (PTC BS) and after (PTC AS) surgery in PTC patients, from control groups with BG, and (**d1**) from vesicles isolated from the MDAT32 cell line culture medium.

EVs were isolated from the plasma of 12 patients (BG: aged between 54 and 71 years old, 4 males and 8 females; PTC: aged between 32 and 65 years old, 12 females) and from the cell culture medium of the MDAT32 cell line. NTA, DLS, and STEM were performed for the biophysical and morphological characterization of EVs. With the NTA measurements, the purified EVs showed a size distribution in the expected range of 100–124 nm, typical of EVs (Figure 1). DLS measurements were performed to further characterize the particle size distribution of EVs. From the size distribution, it was confirmed that the EVs had a size range of 120–130 nm (Figure 1). Since morphology is another important property that distinguishes EVs, the STEM method was used to define the morphology and shape of the EVs obtained from all groups studied. The images demonstrated the typical round-shape morphology of EVs (Figure 1).

Isolated EVs were thoroughly characterized, using Western blotting, to detect specific EV markers such as ALIX and CD81. As expected, this analysis confirmed: (a) The presence of CD81 and Alix in EVs isolated from both the plasma samples of patients with PTC, subjects with BG, and cell culture medium of the MDAT32 cell line; and (b) the absence of these markers in the corresponding cell lysates, further validating the successful isolation of EVs (Figure 1).

To assess the purity of isolated EVs, the absence of calnexin was also demonstrated. Calnexin is an endoplasmic reticulum protein that is cell-specific and is expected to be absent in EVs. As expected, this analysis confirmed: (a) The absence of calnexin in the EVs and (b) the presence of calnexin in the cell lysates, confirming the integrity and purity of our EV preparations (Figure 1). The images presented in Figure 1 are representative of the data obtained from EVs isolated from the plasma of patients with PTC, subjects with BG, and the MDAT32 cell line. These results underscore the efficacy of our isolation method in obtaining pure EVs, which were subsequently subjected to further investigations, including Real-Time PCR, to determine the levels of specific miRNAs. This characterization is crucial as it provides a strong foundation for the subsequent molecular analyses aimed at identifying potential biomarkers for PTC.

### 3.2. Evaluation of microRNAs Levels in EVs

**Figure 2:**
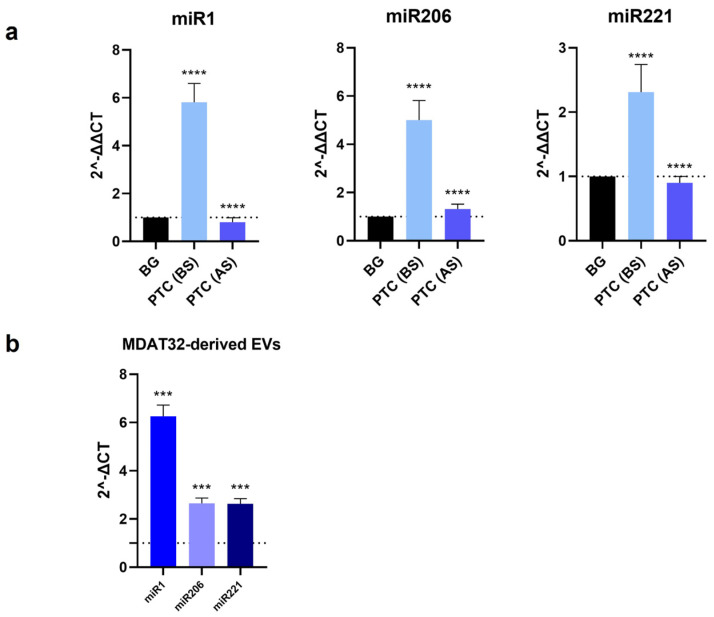
Measurement of miRNAs levels in EVs. Histograms showing the levels of miR-1-3p, miR-206, miR-221-3p in EVs from BG (control patients) and PTC patients before surgery (BS) and after surgery (AS). Statistical analysis was performed using an unpaired two-tailed student *t*-test (**** *p* < 0.0001). Mean values ± SD from 12 different experiments (**a**). Histograms showing the levels of miRNAs isolated from MDAT32-derived EVs. Data were analyzed by one-way ANOVA with Tukey’s multiple comparisons test (*** *p* < 0.001). Mean values ± SD from 10 different experiments (**b**).

To identify potential miRNA-based biomarkers for PTC, we evaluated the levels of specific miRNAs isolated from both MDAT32-derived EVs and EVs isolated from plasma samples of patients with PTC and BG (before and after surgery). The results showed that the levels of miR-1-3p, miR-206, and miR-221-3p within plasma-derived EVs were significantly higher in patients with PTC than in BG (Figure 2a). In patients with PTC, following surgery, there was a decrease in the levels of all three miRNAs analyzed (Figure 2a). Data were analyzed using the comparative CT method (2^–δδct^) with BG serving as the reference sample [31]. The levels of miR-1-3p, miR-206, and miR-221-3p in MDAT32-derived EVs were also significantly higher (Figure 2b). Data are presented as individual 2^ΔCT values for miRNA expression profiling, reflecting the relative expression levels without a reference sample [31].

### 3.3. miRNA Targets Prediction for Thyroid Cancer-Associated Genes

The results obtained for each miRNA analyzed are presented in Table 2. The Total Context Score on TargetScan is a comprehensive metric used to evaluate the likelihood that a specific miRNA will regulate a target gene, identifying a potential miRNA binding site in regulatory sequences of a gene. This score integrates various factors that influence the effectiveness of miRNA-mediated gene regulation, including complementarity between the seed region of the miRNA and the binding site on the target mRNA, location and accessibility of the binding site (such as within the 3′ untranslated region), and number of binding sites. When the total context score is negative and low, the probability that miRNA affects target gene expression is higher [32]. Thus, the Total Context Score serves as a critical predictive tool for identifying biologically relevant miRNA-target interactions, with lower scores suggesting a higher probability of significant regulatory impact by the miRNA on its target gene. The bioinformatic analysis shows the genetic targets of the studied miRNAs, with a lower Total context score, that may contribute to the development and progression of thyroid cancer.

## 4. Discussion

The incidence and morbidity of thyroid cancer have increased over the past decades [33]. Consequently, the search for useful biomarkers is becoming more and more necessary for developing accurate diagnostic and patient-monitoring tools. There is growing evidence showing the involvement of miRNAs in thyroid cancer [7,18,25]. Among the miRNAs frequently dysregulated in thyroid cancer, miR-1-3p and miR-206 are particularly important because of their role in regulating Hsp60, a member of the CS involved in the progression of PTC. To date, the diagnostic accuracy of miRNAs in PTC has not been conclusively established, demanding further studies. The aim of the present study was to explore whether the levels of three miRNAs (miR-1-3p, miR-206, and miR-221-3p) in EVs isolated from the plasma (liquid biopsy) of patients with PTC would be consistently elevated and, thus, serving as diagnostic biomarkers. Additionally, we explored the potential mechanisms involving miR-1-3p and miR-206 in PTC through bioinformatics analysis. Patients with PTC were compared with patients with BG before and after the surgical removal of the thyroid. In this study, we selected BG patients as controls based on our previous research, which investigated tissue levels of heat shock proteins [20]. BG is a pathological condition characterized by diffuse enlargement of the thyroid gland or the presence of multiple localized nodules. Its etiology involves genetic and environmental factors, particularly iodine deficiency. Given the distinct microscopic morphology of BG, which includes macro- and micro-follicles surrounded by follicular cells, compared to the malignant features of PTC, analyzing the levels of selected miRNAs in plasma EVs from BG patients may provide valuable insights for differential diagnosis through a minimally invasive approach. EVs were characterized by dynamic light scattering (DLS) and STEM and nanoparticle tracking analyses to verify the presence of vesicles with a size of 100–120 nm. Western blotting was performed to detect specific EV markers like CD81 and Alix. We focused on miR-221-3p because it is deregulated in thyroid carcinomas and promotes cancer cell migration and invasion [26,34]. In addition, we focused on miR-1-3p and miR-206, which are predicted to regulate Hsp60 expression, a chaperonin involved in the development of thyroid cancer [19,20,21]. Previous works showed that Hsp60 is elevated in carcinoma tissues [35,36,37,38,39] and is found at high levels in plasma from subjects with PTC [20]. The MDAT32 cell line also showed high levels of Hsp60.

The levels of miR-1-3p, miR-206, and miR-221-3p were significantly higher in patients with PTC before surgery (BS) compared to control patients (BG). Also, the levels of miRNAs before surgery were higher than the levels after surgery (AS). Therefore, the data indicate that these miRNAs, which were chosen for this study because they might be involved in thyroid tumorigenesis [13,20], are promising biomarkers for the diagnosis and monitoring of PTC patients.

Bioinformatics analysis [40] revealed, among the miRNA target genes examined, the presence of genes involved in tumorigenesis (e.g., MET, HDAC4, VEGFA, TP53BP2), in cell cycle regulation (e.g., CCND2, CDKN1B, CDKN1C), and in apoptosis (e.g., CAAP1, BCL2L11).

Specifically, MET encodes a receptor tyrosine kinase that is implicated in cell proliferation and frequently overexpressed in various cancers, and HDAC4 (Histone deacetylase 4) influences chromatin structure and regulates cancer proliferation and progression. HDAC4 is known to interact with various transcription factors, influencing cancer progression and potentially serving as a therapeutic target [41,42,43]. CCND2 is a member of the cyclin family that binds to cyclin-dependent kinases (CDKs), promoting cell progression from the G1 to S phase of the cell cycle. The dysregulation of this process can lead to uncontrolled cell proliferation, a common feature of cancer cells [44]. In contrast, CDKN1B and CDKN1C are cyclin-dependent kinase (CDK) inhibitors, p27^Kip1^ and p57^Kip2^ respectively, that usually act as tumor suppressors, but their regulation is altered during tumor processes, leading to unchecked cellular proliferation [45].

Targets also included genes that influence tumor diffusion via metastasis, such as TAGLN2 involved in Transgelin 2 protein expression. Transgelin is a protein involved in cytoskeletal organization and cell motility, the alteration of which can affect tumor cell migration [46]. Furthermore, chaperonins protect tumor cells from stress, allowing them to survive and proliferate [21], and the bioinformatics analysis revealed HSPD1 (the molecular chaperone or chaperonin type I Hsp60) and CCT5 subunit (a component of the type II CCT chaperonin) among the target genes of the miRNAs examined. As stated above, the molecular chaperone Hsp60 is involved in the proper folding of mitochondrial proteins and in the protection of cells from stress. In cancer, Hsp60 supports tumor cell survival and proliferation under stressful conditions, making it a key player in tumor biology [9,12,20,21,35,36,37,38,39]. CCT5 is involved in the folding of cytoskeletal proteins essential for maintaining cell shape and motility. Its role in cancer is linked to the ability of tumor cells to withstand proteotoxic stress and maintain malignant phenotypes [9,47,48].

These target genes highlight the multifaceted roles of the miRNAs studied in cancer biology, influencing critical pathways that drive tumor development, progression, and resistance to therapy. Therefore, these miRNAs are relevant candidate biomarkers and prospective therapeutic targets in papillary thyroid cancer (PTC) and other malignancies.

This study has some limitations that should be acknowledged at this time, especially to identify future research targets. For example, although the results do bring to light strong biomarker candidates, which was the goal of our study, the determination of their value in clinical practice in general will require testing of many more patients. The study of a larger cohort would be necessary to validate the findings and confirm the potential of miR-1-3p, miR-206, and miR-221-3p as biomarkers for papillary thyroid cancer (PTC). Also, plasma extracellular vesicles (EVs) do provide a minimally invasive approach for biomarker detection and quantification, but the variability in EV isolation and analysis methods must be carefully taken into account in translating the findings to clinical practice. The discovery of biomarkers typically requires a two-step process: an initial exploratory analysis to identify differentially expressed genes (as presented here), followed by a validation step in many patients. Our exploratory study did demonstrate that there are differences in the abundance of the tested miRNAs between the control and cancer patients, and that the differences are consistent and marked to the extent of meriting further investigation. Larger cohorts and additional validation studies will be necessary to establish these miRNAs as reliable biomarkers for PTC in general clinical practice.

Despite these limitations, the study has several strengths. We leveraged a robust and well-characterized experimental approach, including the analysis of miRNAs within EVs, which offers protection from circulating RNAses and provides a stable source of potential biomarkers. Furthermore, our integration of bioinformatics analysis to predict target genes of the miRNAs studied adds a deeper understanding of the molecular mechanisms involved in PTC tumorigenesis. The study’s focus on miRNAs associated with the chaperone system, particularly miR-1-3p and miR-206, underscores their potential as specific biomarkers linked to Hsp60 expression, a protein implicated in PTC development [9,20]. These findings contribute to the growing body of evidence supporting the role of EV-associated miRNAs in cancer diagnosis and patient monitoring, highlighting their potential utility in the differential diagnosis of thyroid nodules.

## 5. Conclusions

In conclusion, the findings of this study align with the increasing body of literature suggesting that microRNAs (miRNAs) are implicated in tumorigenesis, including thyroid cancer [49]. The results indicate that miR-1-3p, miR-206, and miR-221-3p, present in extracellular vesicles (EVs) isolated from the plasma of patients with papillary thyroid carcinoma (PTC), are significantly elevated before surgery compared to controls and show a notable decrease post-surgery. These observations highlight the potential of these miRNAs as diagnostic biomarkers and tools for monitoring disease progression in PTC. Additionally, bioinformatics identified for the three miRNAs studied several target genes associated with cancer-related processes, such as tumorigenesis, cell cycle regulation, and apoptosis. These insights underscore the necessity for further investigation into the clinical utility of the miRNAs examined in this work in the context of thyroid cancer.

## Figures and Tables

**Table 1:** miRNA PCR primers used for Real-Time PCR Assays (QIAGEN, Hilden, Germany).

miRNA	Code	Sequence	Dilution
miR-16-1-3p	YP00206012	MIMAT0004489: 5′CCAGUAUUAACUGUGCUGCUGA	1:10
miR-1-3p	YP00204344	MIMAT000416: 5′UGGAAUGUAAAGAAGUAUGUAU	1:10
miR-206	YP00206073	MIMAT0000462: 5′UGGAAUGUAAGGAAGUGUGUGG	1:10
miR-221-3p	YP00204532	MIMAT0000278: 5′AGCUACAUUGUCUGCUGGGUUUC	1:10

**Table 2:** Thyroid cancer-associated genes that are predicted targets of miR-1-3p, miR-206, and miR-221-3p miRNA.

microRNAs	Target Gene	Gene Name	Total Context Score ^1^
miR-1-3p	MET	met proto-oncogene	−0.27
	HDAC4	histone deacetylase 4	−0.29
	CCND2	cyclin D2	−0.53
	TAGLN2	transgelin 2	−0.85
	CAAP1	caspase activity and apoptosis inhibitor 1	−0.48
	HSPD1	heat shock 60kDa protein 1 (chaperonin)	−0.35
miR-206	NOTCH3	notch receptor 3	−0.23
	VEGFA	vascular endothelial growth factor A	−0.20
	CCND2	cyclin D2	−0.53
	CDON	cell adhesion-associated, oncogene regulated	−0.51
	MET	met proto-oncogene	−0.27
	CXCR4	chemokine (C-X-C motif) receptor 4	−0.38
	HSPD1	heat shock 60kDa protein 1 (chaperonin)	−0.35
miR-221-3p	CDKN1B	cyclin-dependent kinase inhibitor 1B (p27, Kip1)	−1.05
	TIMP3	TIMP metallopeptidase inhibitor 3	−0.20
	RECK	reversion-inducing-cysteine-rich protein with kazal motifs	−0.26
	CCT5	chaperonin containing TCP1, subunit 5 (epsilon)	−0.69
	TP53BP2	tumor protein p53 binding protein, 2	−0.54
	CDKN1C	cyclin-dependent kinase inhibitor 1C (p57, Kip2)	−0.35
	BCL2L11	BCL2-like 11 (apoptosis facilitator)	−0.42

^1^ The total context score is an indicator of the efficiency of target gene regulation by miRNAs. A negative and low Total Context Score indicates a higher probability of miRNA affecting target gene expression [32].

## Data Availability

The original contributions presented in the study are included in the article, further inquiries can be directed to the corresponding author/s.
